# Metastatic site-specific polarization of macrophages in intracranial breast cancer metastases

**DOI:** 10.18632/oncotarget.9445

**Published:** 2016-05-18

**Authors:** Nora Rippaus, David Taggart, Jennifer Williams, Tereza Andreou, Heiko Wurdak, Krzysztof Wronski, Mihaela Lorger

**Affiliations:** ^1^ Institute of Cancer and Pathology, University of Leeds, St. James's University Hospital, LS9 7TF Leeds, UK; ^2^ Geneflow Ltd, Elmhurst, Lichfield, Staffordshire WS13 8EX, UK

**Keywords:** metastasis-associated macrophages, tumor-associated macrophages, breast cancer brain metastases, dural metastases, lymphotoxin β

## Abstract

In contrast to primary tumors, the understanding of macrophages within metastases is very limited. In order to compare macrophage phenotypes between different metastatic sites, we established a pre-clinical mouse model of intracranial breast cancer metastasis in which cancer lesions develop simultaneously within the brain parenchyma and the dura. This mimics a situation that is commonly occurring in the clinic. Flow cytometry analysis revealed significant differences in the activation state of metastasis-associated macrophages (MAMs) at the two locations. Concurrently, gene expression analysis identified significant differences in molecular profiles of cancer cells that have metastasized to the brain parenchyma as compared to the dura. This included differences in inflammation-related pathways, NF-kB1 activity and cytokine profiles. The most significantly upregulated cytokine in brain parenchyma- versus dura-derived cancer cells was Lymphotoxin β and a gain-of-function approach demonstrated a direct involvement of this factor in the M2 polarization of parenchymal MAMs. This established a link between metastatic site-specific properties of cancer cells and the MAM activation state.

## INTRODUCTION

Breast cancer is the second leading cause of cancer deaths in women and it is estimated that over 90% of these deaths are caused by metastasis [1, 2]. Breast cancer commonly metastasizes to the lungs, bone, liver, and the central nervous system (CNS) [3]. CNS metastases occur in ~15% of breast cancer patients, with a significantly higher proportion in those with triple negative and HER2-positive breast cancer (30–50%). Due to the lack of effective therapies, CNS metastases are associated with a particularly poor prognosis and the median survival time after diagnosis is only 3–24 months [4]. CNS metastases typically develop within the brain parenchyma, the dura or the leptomeninges [5]. Notably, patients commonly develop metastases at multiple extracranial and/or intracranial sites and these metastatic lesions differ in molecular characteristics of cancer cells [6, 7], and in their tumor microenvironment [8, 9]. This leads to variable responses to therapy between cancer lesions at different anatomical locations, complicating the treatment of metastatic cancer [10–13]. It is therefore important to gain a better understanding of molecular and cellular differences between metastatic sites.

Tumor-associated macrophages (TAMs) are an important component of the tumor microenvironment. They can adopt different activation/polarization states that represent a continuum between the two extremes of anti-tumorigenic M1 and pro-tumorigenic M2-like macrophages, with TAMs mainly being skewed towards the M2 state [14–17]. Varied tumor-promoting functions that have been demonstrated for TAMs and their role in the modulation of responses to therapies depend on their activation state [14–18]. While the majority of studies have focused on TAMs at the primary tumor site [16], more recently a distinct population of so-called metastasis-associated macrophages (MAMs) has been implicated in cancer cell extravasation and subsequent growth in lung metastasis [19–22]. These studies also showed that macrophages infiltrating the primary tumor and metastases differ in their origin and in their phenotypes [20]. However, whether macrophage phenotypes also differ between different metastatic locations remains unexplored.

To address this knowledge gap, we established a clinically relevant model of simultaneous dural and parenchymal brain metastasis, which are observed in 23% of breast cancer patients with CNS involvement [5]. This approach enabled the characterization of MAMs at these two frequently co-occurring metastatic locations. Our objectives were to identify potential metastatic site-specific differences in MAM activation states and to investigate how the MAM phenotypes are regulated, with a focus on the cross-talk between MAMs and cancer cells.

## RESULTS

### Pre-clinical models of intracranial breast cancer metastases with simultaneous involvement of brain parenchyma and the dura

**Figure 1:**
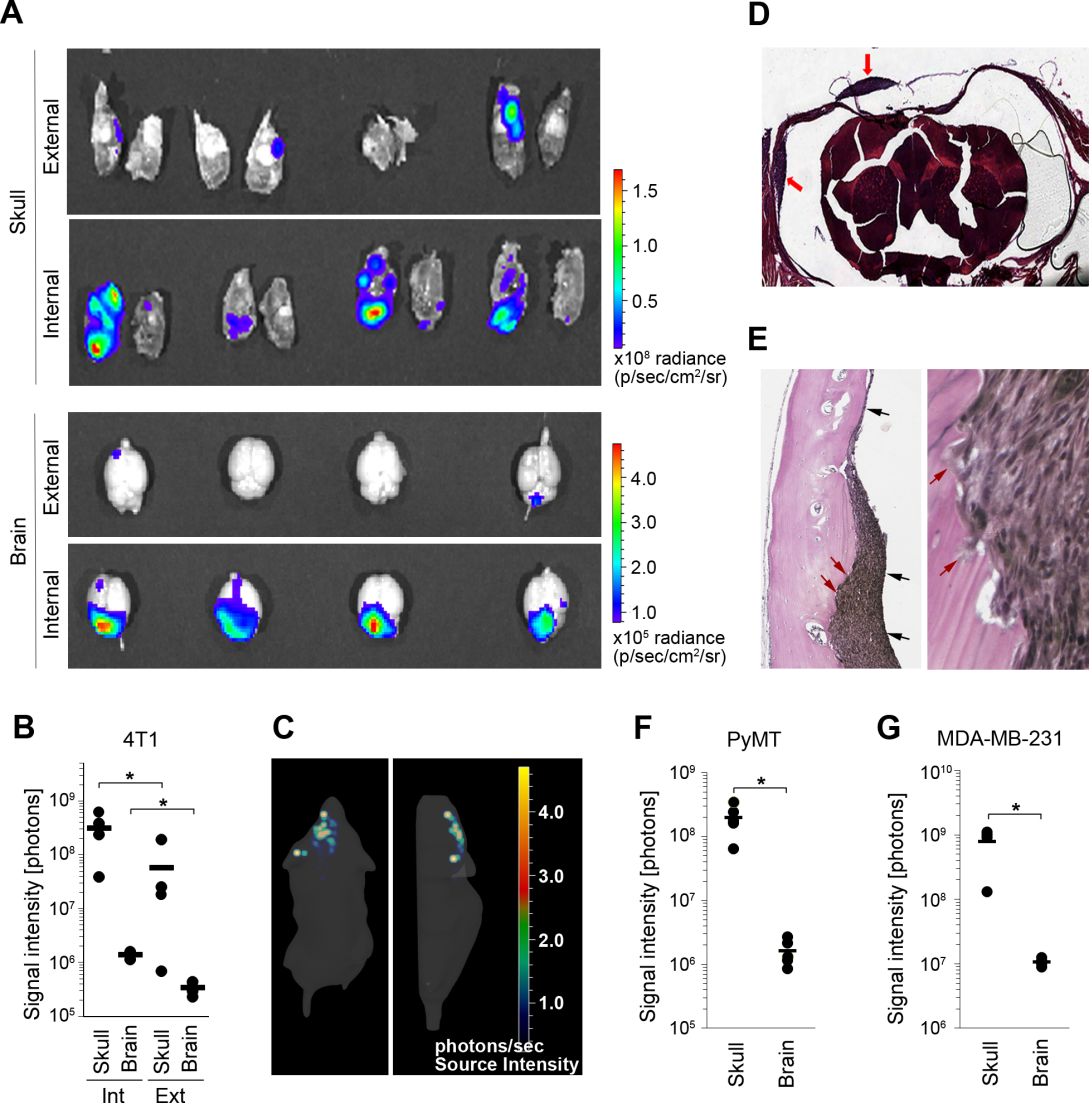
4T1 breast cancer model with simultaneous metastasis to the brain parenchyma and the dura (**A**) The distribution of metastatic lesions 10 days after administration of Fluc-tagged 4T1 cancer cells into the external or internal carotid artery was analyzed by *ex-vivo* bioluminescence imaging of the brain parenchyma (brain) and the skull/dura (skull). (**B**) Quantification of bioluminescence signal shown in A. Int: internal; Ext: external; (**C**) 3D *in vivo* bioluminescence imaging of cancer cells 10 days after their administration into the internal carotid artery. Dorsal (left) and side view (right) are shown. The majority of cancer lesions are localized at the top of the head, suggesting predominant tumor burden at the skull/dura. (**D**) H&E staining of coronal head sections containing dural metastases (red arrows). (**E**) Verhoeff-Van Gieson staining of dural metastases. Dura mater is marked with black arrows (left image). Invasion of cancer cells into the skull is marked with red arrows (left and right image). (F and G) Distribution of cancer lesions between the skull/dura and the brain parenchyma was analyzed by *ex vivo* bioluminescence imaging at 16 and 45 days post-cancer cell injection into the internal carotid artery using PyMT (**F**) and MDA-MB-231 cancer cell lines (**G**), respectively. Statistical significance in B, F and G was determined using two-tailed Student's *T*-test with unequal variance (*p* ≤ 0.05); *n* = 4.

The triple-negative breast cancer subtype is associated with a high rate of intracranial metastases [4]. Therefore, we used the triple-negative 4T1 breast cancer cell line to identify an experimental approach for the most efficient colonization of multiple intracranial sites. We compared intracranial colonization after the administration of F-luc-tagged 4T1 cancer cells into the external versus internal carotid artery of BALB/c mice (these two arteries have been previously shown to supply distinct intracranial locations [23]). At 10 days post-administration of cancer cells, the brain parenchyma and the skull with dural membrane (e.g. dura mater; dura) were isolated. Tumor burden at different intracranial sites was quantified by *ex vivo* bioluminescence imaging. Comparison of experimental groups that received cancer cells via the external versus internal carotid artery showed that the latter resulted in a significantly higher tumor burden within the brain (4-fold) as well as the skull/dura (150-fold) (Figure 1A, 1B). Furthermore, both administration routes resulted in a predominant (> 92% on average) skull/dura-associated tumor burden (Figure 1A, 1B). This was confirmed by 3D bioluminescence imaging of mice receiving cancer cells via the internal carotid artery (Figure 1C). Quantification of GFP-tagged cancer cells within brain parenchyma and the dura by flow cytometry further confirmed significantly higher numbers of cancer cells at the dura (Supplementary Figure S1C). Notably, a similar distribution of bioluminescence signal was observed when a 10-fold lower number of cancer cells (1 × 10^4^) was administered into the internal carotid artery (Supplementary Figure S1A), suggesting that this pattern of dissemination doesn't depend on the number of injected cancer cells. Due to the higher colonization efficiency, the internal carotid artery was used for administration of cancer cells in all subsequent experiments.

To determine the nature of skull/dura-associated metastases, we first performed microscopic examination. This revealed metastatic foci that were attached to the dural membrane, and identified these lesions as dural metastases. Histology of coronal head sections confirmed the location of metastatic lesions between the skull and the dura mater (Figure 1D, 1E). The dural membrane in these lesions appeared mostly intact and occasional cancer cell infiltration into the skull was detected in larger lesions (Figure 1E). In addition to dural metastases, lesions within the skull could also be detected by histology (Supplementary Figure S1B).

Importantly, injection of two further breast cancer cell lines - murine carcinoma PyMT (C57Bl6 mice) and the human triple-negative cancer cell line MDA-MB-231 (CB17/scid mice) - into the internal carotid artery reproducibly generated both dural and parenchymal metastases (Figure 1F, 1G and Supplementary Figure S1D, S1E). Skull/dura-associated tumor burden was again significantly higher compared to the parenchymal tumor burden. In summary these data demonstrated that simultaneous metastasis to the dura and brain parenchyma can be reliably modeled following the administration of different breast cancer cell lines into the internal carotid artery.

### Distinct inflammatory tumor microenvironments in dural and parenchymal brain metastases

**Figure 2:**
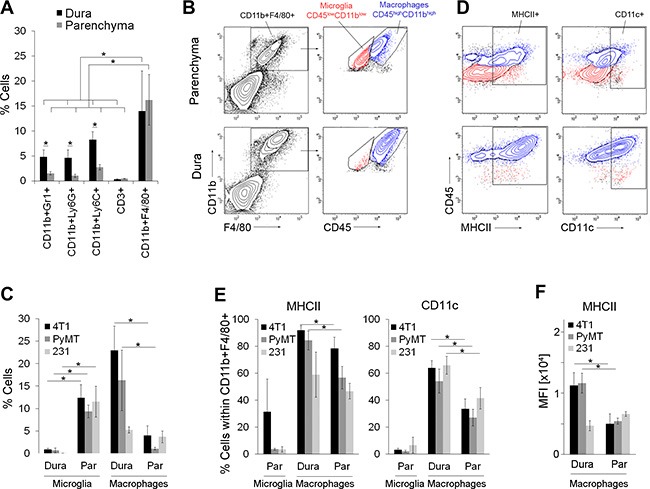
Inflammatory tumor microenvironment in dural and parenchymal brain metastases (**A**) Infiltration of immune cells into dural (dura) and parenchymal 4T1 cancer lesions (parenchyma) in intracarotid artery model; *n* = 5. (**B**) Representative flow cytometry analysis of microglia and macrophages within parenchymal (top) and dural lesions (bottom) in 4T1 breast cancer model. Microglia were identified as CD45^low^CD11b^low^ cells (red) and macrophages as CD45^high^CD11b^high^ cells (blue) within the CD11b^+^F4/80^+^ gate. (**C**) Quantification of microglia and macrophages in 4T1, PyMT and MDA-MB-231 (231) models based on the flow cytometry analysis shown in Figure 2B, Supplementary Figure S5A–S5B; *n* = 4. (**D**) Representative flow cytometry analysis of MHCII and CD11c expression in the microglia (red) and macrophages (blue) within parenchymal (top) and dural (bottom) 4T1 metastases. The contour plots were gated on the CD11b^+^F4/80^+^ population shown in B. (**E**) Quantification of MHCII^+^ and CD11c^+^ cells within the microglia and macrophage populations in 4T1, PyMT and MDA-MB-231 models based on the flow cytometry analysis shown in Figure 2D, Supplementary Figure S5A–S5B; *n* = 4. (**F**) Mean fluorescent intensity (MFI) for MHCII expression in dural and parenchymal MAMs; *n* = 4. Statistical significance in A, C, E and F was determined using one-tailed Student's *T*-test with unequal variance (*p* ≤ 0.05). Error bars represent standard deviations (SD).

Models of simultaneous dural and parenchymal brain metastases allowed us to compare the inflammatory tumor microenvironment, including the MAMs, at these two co-occurring metastatic locations. Inflammatory cells in dural and parenchymal lesions established after the injection of 4T1 cells into the internal carotid artery were analyzed by flow cytometry. The infiltration of myeloid-derived suppressor cells (MDSCs; CD11b^+^Gr1^+^), granulocytes (CD11b^+^Ly6G^+^) and monocytes (CD11b^+^Ly6C^+^) into dural metastases was significantly greater (3 to 4.5-fold) than in parenchymal lesions (Figure 2A and Supplementary Figure S2). Only a very low infiltration rate of T-cells (CD3e^+^) was detectable at either location. Microglia/macrophages (CD11b^+^F4/80^+^) infiltrated dural and parenchymal metastases at a similar rate and were the most abundant immune cell population at both sites. These findings were confirmed by immunofluorescence (Supplementary Figure S3A).

Similar to 4T1-derived cancer lesions, CD11b^+^F4/80^+^ cells were also the most abundant infiltrating cell population within both dural and parenchymal brain metastases derived from the PyMT and MDA-MB-231 cancer cell lines (Supplementary Figure S3B), demonstrating that predominant infiltration of microglia/macrophages is cancer cell line-independent.

We further separated putative microglia from macrophages by flow cytometry based on CD11b and CD45 expression levels as previously demonstrated [24–27]. This revealed that microglia (F4/80^+^CD11b^low^CD45^low^) were the predominant cell population in parenchymal metastases, while they were barely detectable in dural metastases (Figure 2B, 2C and Supplementary Figure S4A). MAMs (F4/80^+^CD11b^high^CD45^high^) could be detected in parenchymal and dural metastases, with a significantly higher proportion in the latter in both immunocompetent models (4T1 and PyMT-derived) (Figure 2B, 2C and Supplementary S4A). Notably, dural cancer lesions were micro-dissected from the skull and the dura prior to analysis, and therefore these samples contained only metastases-associated macrophages. In contrast to the metastases-bearing brain parenchyma, the F4/80^+^CD11b^high^CD45^high^ cell population was hardly detectable in naïve brains (below 0.5%; Supplementary Figure S4C), demonstrating the association of CD45^high^ macrophages with metastases.

Among other differences, pro-inflammatory (anti-tumorigenic) M1 macrophages are associated with increased antigen-presenting cell (APC) function as compared to the anti-inflammatory (pro-tumorigenic) M2 macrophages [29]. Thus, to compare MAMs and microglia between dural and parenchymal metastases, we analyzed the expression of MHCII and CD11c, markers that have been previously associated with the APC phenotype in microglia/macrophages [30, 31]. In line with studies from glioma and other non-cancerous CNS disorders [31, 32], flow cytometry analysis showed that only a small percentage of parenchymal microglia expressed MHCII or CD11c independent of the breast cancer model (range 3–30% and 2–6.5%, respectively; Figure 2D–2E, Supplementary Figure S4B, Supplementary Figure S5A–S5B). In the 4T1 and PyMT models, the proportion of MHCII-expressing MAMs was significantly higher in dural versus parenchymal metastases (84–92% versus 56–78%). Moreover, MHCII^+^ dural MAMs expressed 2-fold higher levels of MHCII compared to the parenchymal MAMs (Figure 2F). Similarly, the proportion of CD11c-expressing MAMs was approximately 2-fold higher in dural versus parenchymal metastases and this difference was significant across all 3 cancer models (Figure 2D–2E, Supplementary Figure S4B, Supplementary Figure S5A–S5B). In summary, this suggested that dural MAMs have a higher antigen presenting potential compared to the MAMs within parenchymal brain metastases.

### Cancer cells that have metastasized to the brain parenchyma and the dura differ in inflammation-related molecular signatures

Metastatic cancer cells are known to develop organ-specific molecular signatures [6, 7]. We therefore compared cancer cells that have metastasized to the dura and brain parenchyma to identify potential differences and to investigate to what extent the molecular profiles of cancer cells are linked to the site-specific MAM phenotypes.

**Figure 3:**
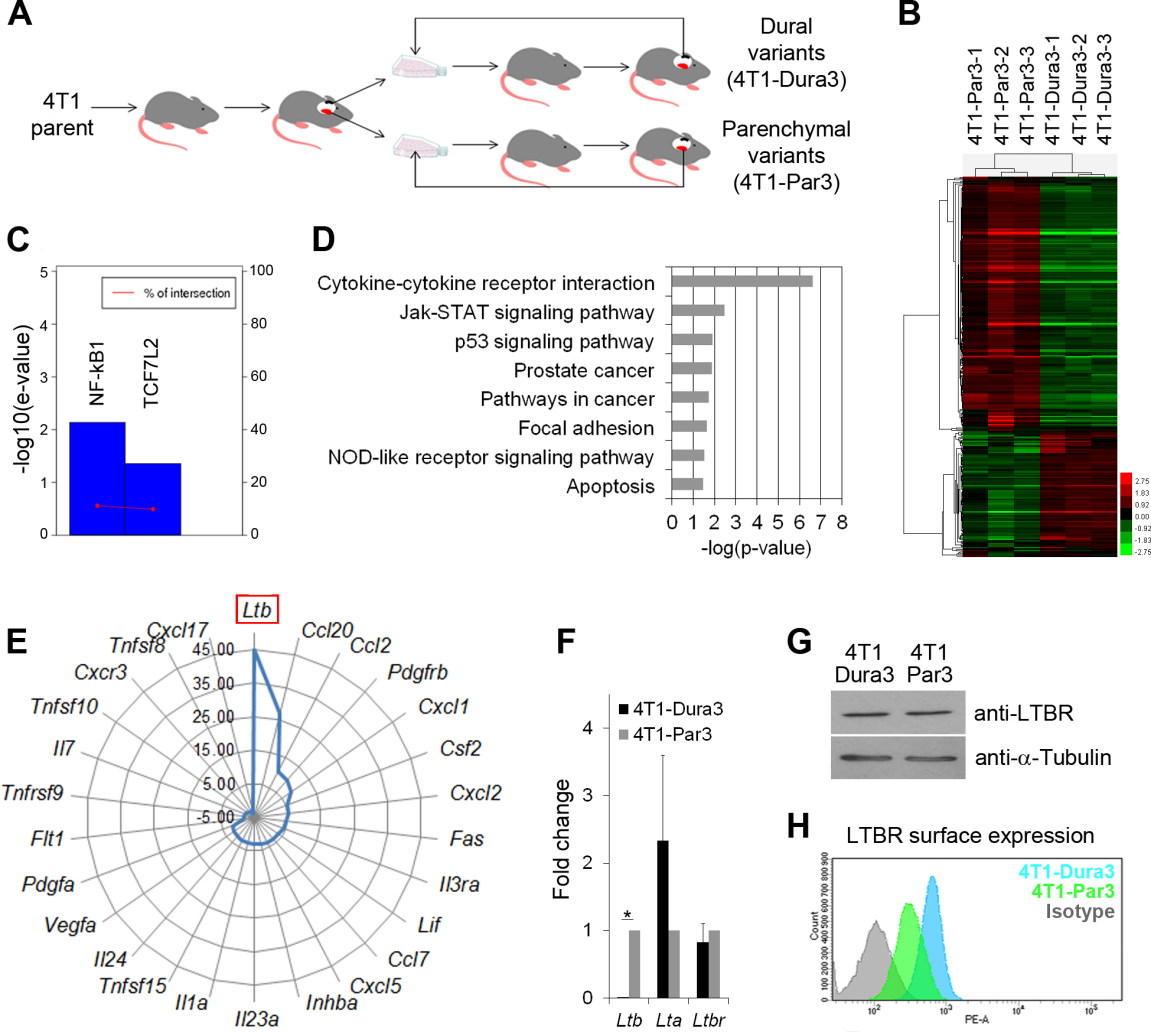
4T1 cancer cells evolve distinct molecular profiles after they have metastasized to the dura versus brain parenchyma (**A**) Experimental outline for the *in vivo* selection of 4T1 cancer cell variants with site-specific characteristics. Dural cancer lesions are illustrated in black and parenchymal lesions in red. (**B**) Heat map and hierarchical clustering of genes differentially expressed between the 4T1-Par3 and 4T1-Dura 3 cancer cell variants. (**C**) Upregulation of the NF-kB1 and TCF7L2 (TCF4) transcription factor activity in parenchymal versus dural 4T1 cell variants. E-value scores and intersection percentage for significantly inhibited transcription factors (e-value < = 0.05) in dural versus parenchymal cell variants were determined using TFactS software. (**D**) Summary of the signaling pathways that were differentially regulated between the 4T1-Par3 and 4T1-Dura 3 cancer cell variants. (**E**) Graphic summary of the cytokines that were significantly upregulated in parenchymal versus dural 4T1 cancer cell variants. (**F**) Quantification of *Ltβ, Ltα, and Ltβr* mRNA by qRT-PCR. Statistical significance was determined using two-tailed Student's *T*-test with unequal variance (*p* ≤ 0.05). Error bars represent SD. (**G**) Analysis of total LTβR protein expression in whole cell lysates by Western blot. One out of three independent experiments is shown. (**H**) LTβR surface expression (MFI) is reduced in 4T1-Par3 versus 4T1-Dura3 cell lines as quantified by flow cytometry.

To enrich for cancer cells with site-specific characteristics and allow for their robust analysis, 4T1 cancer cells were isolated from the dura and brain parenchyma after 3 consecutive rounds of site-specific *in vivo* selection (4T1-Dura3 and 4T1-Par3 cell variants, respectively) (Figure 3A). Biological triplicates isolated from each location were subjected to whole transcriptome microarray analysis. Bioinformatics analysis identified a set of 645 unique genes that were differentially regulated between the two intracranial locations. Among these genes, 230 genes were downregulated and 415 genes were upregulated in the parenchyma- versus dura-derived cancer cells (log2 |Fold change| ≥1 and *P* < 0.05). Hierarchical clustering with this gene set confirmed the distinct gene expression profile of 4T1-Dura3 and 4T1-Par3 cancer cell variants (Figure 3B). Differences in expression levels for the most significantly differentially regulated genes were confirmed by qRT-PCR (Supplementary Figure S6A).

Interrogation of transcription factor networks using TFactS software [34] revealed a significantly higher activity of NF-kB1 and TCF7L2 (TCF4) in parenchymal compared to dural cancer cell variants (Figure 3C). Moreover, gene set enrichment analysis identified 8 pathways that were significantly altered between dural and parenchymal cancer cells (Figure 3D and Supplementary Table S1). The most significant difference was found for the “cytokine-cytokine receptor interactions”. Within this pathway, 7 genes were downregulated and 19 genes upregulated in parenchymal versus dural cancer cells (Figure 3E and Supplementary Table S2). In line with the increased NF-kB1 activity in parenchymal cell variants, 12 of the upregulated genes in the parenchymal cancer cells were known NF-kB1 target genes (see http://www.bu.edu/nf-kb and Supplementary Table S2). Notably, several of the cytokines whose expression was upregulated in parenchymal cancer cells (e.g. CCL2, CCL7, CXCL1, CXCL2, CXCL5, CCL20) have been implicated in the attraction of immune cells [35–37]. In addition, Pyrin within the “NOD-like receptor signaling” pathway, which is implicated in the regulation of the inflammasome [38], was upregulated 3.4-fold in parenchymal versus dural cancer cells (Supplementary Table S3).

Most strikingly, the expression level of Lymphotoxin β *(Ltβ)* was ~45-fold higher in parenchymal compared to dural cancer cell variants (Figure 3E) and this was confirmed by qRT-PCR on pooled biological triplicates (from here on referred to as 4T1-Dura3 and 4T1-Par3 cell lines to distinguish them from non-pooled triplicates called 4T1-Dura3 and 4T1-Par3 variants) (Figure 3F). LTβ is a surface-bound cytokine that has been implicated in the regulation of inflammatory microenvironments [39]. Binding of LTβ to the soluble LTα results in the LTα1β2 heterotrimer, which is one of the main ligands for Lymphotoxin β receptor (LTβR) [39]. Analysis of further components of the LTβ pathway in the 4T1 model confirmed comparable expression levels of both *Ltα* and *Ltβr* in the 4T1-Dura3 and 4T1-Par3 cell lines (Figure 3F). Interestingly, despite similar mRNA and protein expression levels (Figure 3F, 3G), LTβR surface expression was decreased in the 4T1-Par3 compared to the 4T1-Dura3 cell line (Figure 3H). This increase in receptor internalization was indicative of its engagement with its ligand [40], suggesting that elevated *Ltβ* expression in 4T1-Par3 cell line results in increase of functional LTα1β2 heterotrimer and likely in increased autocrine LTβR signaling.

In summary, our data demonstrated that cancer cells that have metastasized to the dura and brain parenchyma significantly differ in their inflammation-related molecular pathways and expression of cytokines, which are known regulators of MAM polarization [14, 33].

### Dural and parenchymal MAMs are characterized by distinct activation states

**Figure 4:**
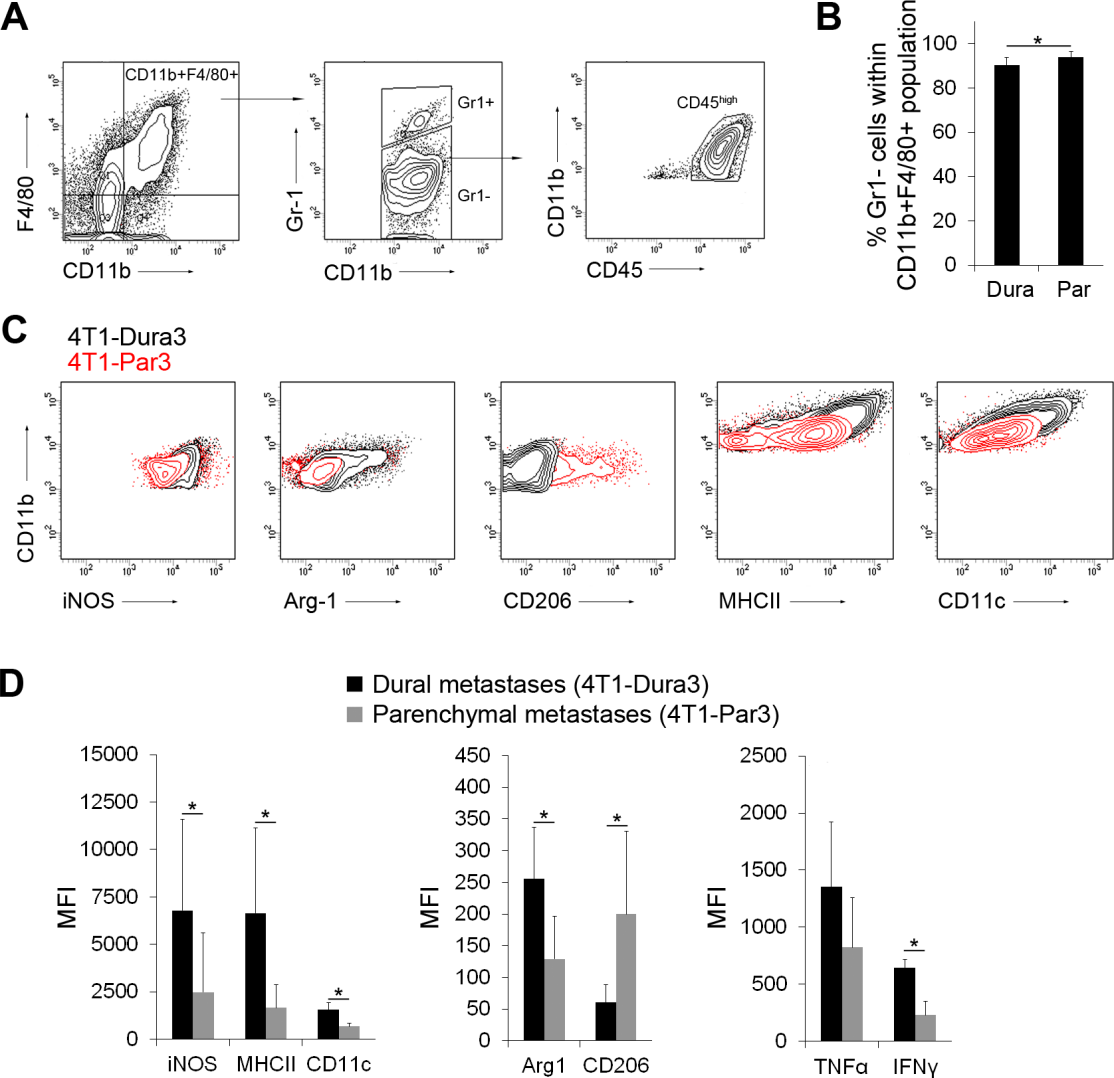
Distinct polarization state of dural and parenchymal MAMs (**A**) Gating strategy for CD11b^+^F4/80^+^Gr1^−^CD45^high^ macrophages. An example of dural lesions is shown. (**B**) Quantification of CD11b^+^F4/80^+^Gr1^−^CD45^high^ MAMs within dural 4T1-Dura3 and parenchymal 4T1-Par3 cancer lesions. (**C**) Representative histograms showing MFI for the expression of M1 (iNOS, MHCII) and M2 (Arginase-1 (Arg-1), CD206) macrophage markers in dural 4T1-Dura3 (black) and parenchymal 4T1-Par3 lesions (red) in intracarotid artery model. (**D**) Quantification of MFI for iNOS, MHCII, CD11c, Arg-1, CD206, TNFα and IFNγ; *n* = 9/11 for dural/parenchymal lesions, respectively. Statistical significance in B and D was determined using one-tailed Student's *T*-test with unequal variance (*p* ≤ 0.05). Error bars represent SD.

To investigate differences between dural and parenchymal MAMs in more detail, we next investigated the 4T1-Par3 and 4T1-Dura3 cell line-derived metastases. We namely hypothesized that cancer cells with enriched site-specific characteristics would result in a more pronounced site-specific polarization of MAMs as compared to cancer cells that have undergone only one round of site-specific selection (e.g. intracranial colonization following the administration of parental 4T1 cancer cells into the carotid artery). To this end, parenchymal brain metastases were established upon the administration of 4T1-Par3 cell line into the internal carotid artery and dural metastases were established upon the injection of 4T1-Dura3 cell line. To exclude Gr1^+^ immature MDSCs and inflammatory monocytes from analysis [14, 20], we defined MAMs as CD11b^+^F4/80^+^Gr1-CD45^high^ cell population. Notably, the vast majority (> 90%) of CD11b^+^F4/80^+^CD45^high^ cells in our models were Gr1- MAMs in both dural and parenchymal brain metastases (Figure 4A, 4B). The expression of APC markers (MHCII, CD11c), anti-tumorigenic M1 macrophage markers (MHCII, iNOS), pro-tumorigenic M2 macrophage markers (mannose receptor CD206, Arginase-1) and pro-inflammatory cytokines (IFNγ, TNFα) in MAMs was quantified by flow cytometry (Figure 4C–4D, Supplementary Figure S7). This revealed significantly lower expression levels of iNOS (2.7-fold), MHCII (4-fold), CD11c (2.3-fold), Arginase-1 (2-fold), IFNγ (2.8-fold), and TNFα (1.6-fold) in parenchymal versus dural MAMs. In contrast, the expression of CD206 was significantly increased (3.3-fold) in parenchymal MAMs. With the exception of Arginase-1, this pattern of marker expression suggested that the parenchymal MAMs are skewed further towards the M2 state compared to dural MAMs [29], and confirmed the metastatic site-specific MAM polarization.

### Site-specific cancer cell characteristics contribute to MAM polarization

In order to determine whether the metastatic site-specific characteristics of cancer cells are causally linked to the MAM phenotypes, we investigated how the 4T1-Par3 cancer lesions instruct MAM polarization in comparison to the parental 4T1 cancer lesions. To exclude the potential impact of microenvironmental differences, both cancer cell lines were implanted into the brain parenchyma. This omitted the metastatic site-specific selection step that cancer cells undergo during the blood-born brain colonization and better preserved the characteristics of parental 4T1 cancer cells. Because dural colonization is possible only via the blood-born route, we were not able to perform an equivalent experiment at the dural site.

**Figure 5:**
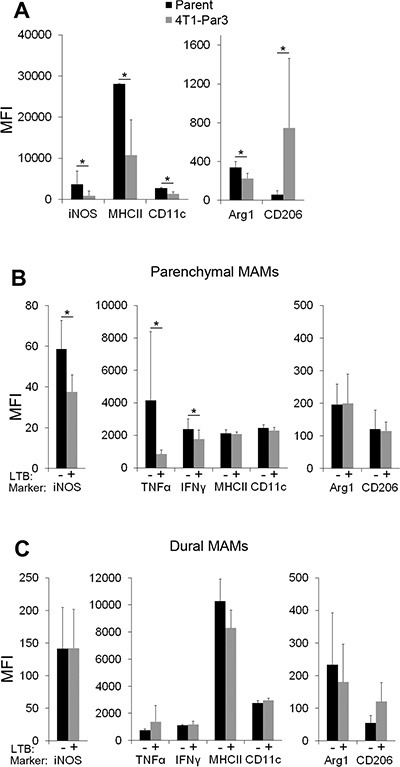
Cancer cell-derived factors contribute to site-specific polarization of parenchymal MAMs (**A**) Expression of macrophage polarization markers in MAMs isolated from the 4T1-Par3 and 4T1 parent-derived cancer lesions established within brain parenchyma after direct intracranial implantation of cancer cells; *n* = 5. (B and C) Expression of macrophage polarization markers in MAMs isolated from parenchymal (**B**) and dural (**C**) brain metastases established from the 4T1-Dura3-LTβ and 4T1-Dura3 control cell lines; *n* = 7. Statistical significance in A–C was determined using one-tailed Student's *T*-test with unequal variance (*p* ≤ 0.05). Error bars represent SD.

Notably, despite the same stromal microenvironment, significant differences were detected in the activation state of MAMs isolated from the 4T1-Par3 as compared to the parental 4T1 cancer lesions growing within the brain parenchyma. In comparison to the 4T1 parent-associated MAMs, the 4T1-Par3-associated MAMs displayed a significantly reduced expression of MHCII, CD11c, iNOS and Arg-1, while CD206 expression was significantly higher (Figure 5A). This demonstrated that cancer cells that underwent metastasis to the brain parenchyma evolve characteristics that enhance the polarization of macrophages towards the M2 state. These findings suggest that colonization of a particular metastatic location leads to the establishment of site-specific characteristics in cancer cells, which subsequently drive the polarization of MAMs.

### Cancer cell-associated Lymphotoxin β is implicated in the site-specific MAM polarization

Having demonstrated a causal involvement of cancer cells in MAM polarization, we next searched for cancer cell-associated factors that could be functionally implicated in this process. LTβ was the most suitable candidate because (i) it was the most significantly differentially expressed cytokine (~45-fold) between the brain parenchyma- and dura-derived 4T1 cancer cell variants; (ii) its role in the regulation of inflammatory microenvironments in secondary lymphoid organs is well established [39]; (iii) recent data linked LTβ to inflammation-induced carcinogenesis [41] and (iv) the agonist-induced activation of LTβR on macrophages has been previously linked to a reduction in their nitric oxide (NO) and cytokine production *in vitro* [42].

To directly test the hypothesis that LTβ is involved in the site-specific MAM polarization, LTβ was stably overexpressed in the 4T1-Dura3 cell line (Supplementary Figure S6B). This was followed by *in vivo* analysis of MAM polarization in dural and parenchymal metastases established after intracarotid administration of the 4T1-Dura3-LTβ and the 4T1-Dura3 control cell line (i.e. 4T1-Dura3 transduced with an empty vector). Notably, the overexpression of LTβ in cancer cells significantly reduced the expression of iNOS, TNFα and IFNγ in parenchymal MAMs by 35%, 79% and 25%, respectively (Figure 5B), implicating LTβ in polarization of MAMs towards the M2 state. In contrast to parenchymal MAMs, the activation state of dural MAMs remained unaffected by the overexpression of LTβ in cancer cells (Figure 5C). This suggested that the regulation of MAM polarization by LTβ is context-dependent and requires microenvironmental cross-talk within brain parenchyma. These findings provided proof-of-concept for a functional link between the metastatic site-specific cytokine expression in cancer cells and the MAM activation state.

## DISCUSSION

TAMs within primary tumors have been studied extensively, while MAMs found in metastases are still poorly understood [14–17]. In our study we discovered that MAMs within intracranial metastases differ between metastatic sites and that those differences are causally linked to the metastatic site-specific cancer cell hallmarks. Due to their role in cancer growth and modulation of therapeutic efficacy [14–18], defining MAM phenotypes at different metastatic locations is important for the development of improved therapies for metastatic disease.

Models of brain metastases employing internal carotid artery as route of cancer cell administration [43–45] have so far focused on analysis of parenchymal brain metastases. Here we showed that this approach also results in dural colonization. This was independent of the cancer cell model, mouse strain, the immune status of the animals, or the number of injected cancer cells. Notably, breast cancer is one of the most frequent cancers associated with dural metastases [46, 47]. A large autopsy study demonstrated dural involvement in 54% and concurrent dural/brain metastases in 23% of breast cancer patients with intracranial disease [5]. Pre-clinical models of dural metastasis were previously lacking, and therefore the model presented here is expected to advance studies of metastases in the dura.

The infiltration of microglia/macrophages into parenchymal brain metastases has been previously reported [45, 48–51]. In continuation of these studies, we here addressed the yolk sac-derived brain-resident microglia [26] and the bone marrow-derived macrophages as two distinct cell populations. Low versus high CD45/CD11b expression levels in microglia as compared to macrophages have been demonstrated in naïve mouse brains, as well as in inflamed brains in mouse models of multiple sclerosis [24–27]. Although deviations from these findings cannot be completely ruled out for brain metastases, the two populations were clearly detectable in our models and the CD45^high^ cells (putative macrophages) were absent from the naïve brains, providing a strong rational for separating microglia from macrophages based on the CD45/CD11b expression levels.

MAMs within the three breast cancer models investigated in this study showed similar site-specific expression levels of MHCII and CD11c, demonstrating a robust and cancer cell line-independent correlation between the metastatic site and MAM phenotype. The only exception was MHCII expression in the MDA-MB-231 model, which may be due to the immunocompromised status of experimental animals as opposed to the immunocompetent mice in 4T1 and PyMT models. Overall, the expression patterns of macrophage polarization markers suggested that parenchymal MAMs are skewed further towards the M2 state as compared to dural MAMs. In this context, concurrently decreased Arginase-1 and iNOS expression in parenchymal versus dural MAMs represents a deviation from the current view that these two enzymes are regulated in opposing directions [14, 15]. However, concurrent regulation of Arginase-1 and iNOS has been demonstrated in MDSCs [14] and our data suggest that the regulation of these enzymes in macrophages may be context-dependent. Notably, M1 and M2 macrophages can be further subdivided into stimuli-dependent phenotypes, demonstrating large phenotypic plasticity [52]. The M1/M2 paradigm is mainly based on well-defined *in vitro* conditions, while *in vivo* the combination of different stimuli can be very complex, thus potentially leading to macrophage phenotypes with co-existing M1 and M2 signatures [53].

Although our study focused on MAMs, it is worth noting that two further immune cell populations implicated in cancer progression – namely neutrophils (Ly6G^+^) and MDSCs (Gr1^+^), were found to be significantly more abundant in dural as compared to parenchymal brain metastases. MDSCs are known to have anti-tumorigenic properties [55] and recent study demonstrated a conversion of neutrophils from pro-tumorigenic into anti-tumorigenic in the course of tumor progression [56]. The functional role of these cell populations in our models, however, remains to be determined.

**Figure 6:**
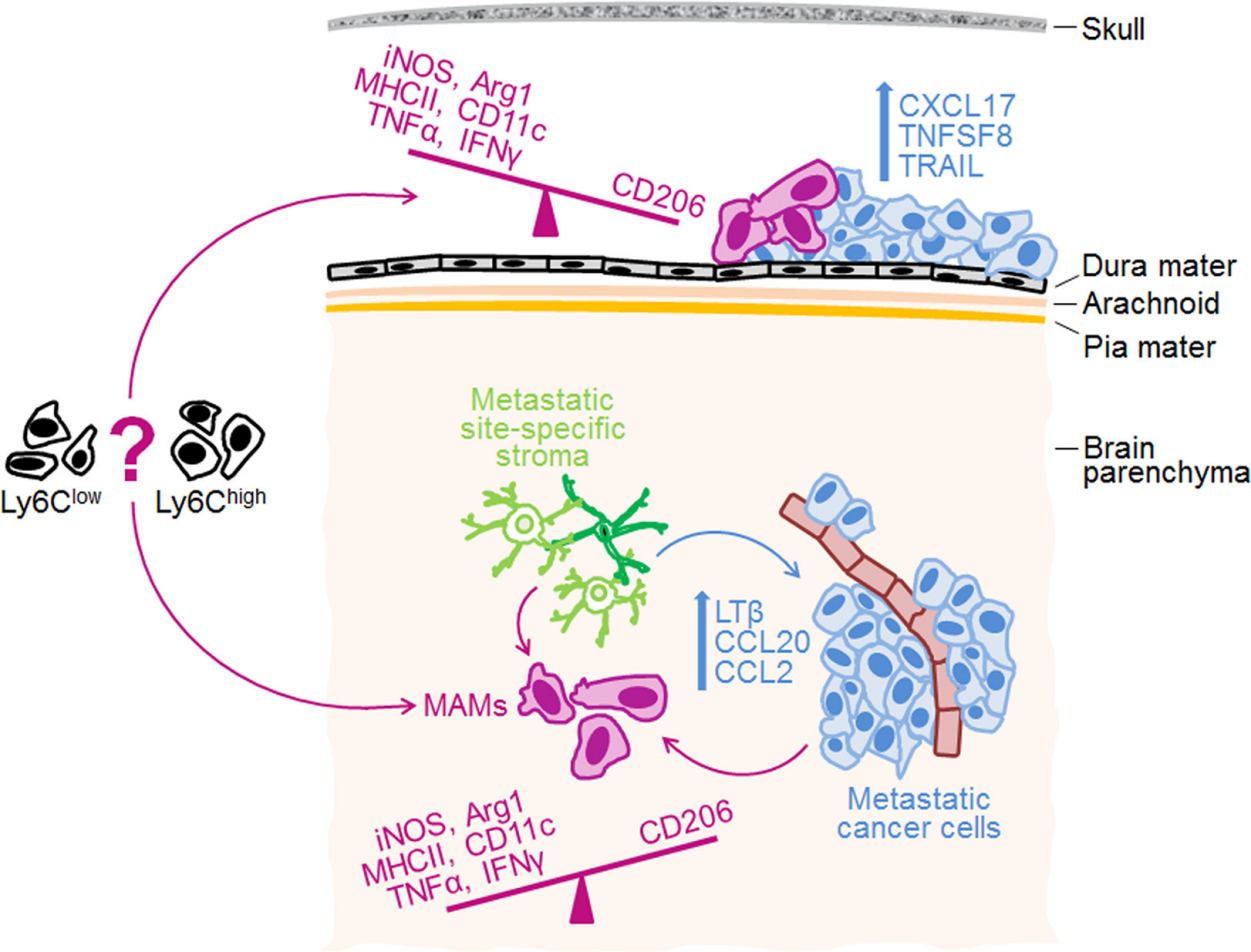
Proposed model for site-specific regulation of MAM phenotypes in intracranial metastases Metastatic site-specific stroma is likely implicated in the establishment of site-specific molecular profiles in cancer cells, which then co-determine MAM phenotypes. The top 3 up-regulated cytokines/chemokines in cancer cells growing at the dura and within brain parenchyma, as well as differences in macrophage polarization markers between the two sites are indicated. Notably, we demonstrated a functional link between LTβ and MAM polarization within the brain parenchyma, while the functions of other cytokines remain to be determined. Based on our findings and evidence from the literature, we propose that MAM activation state is co-determined by the metastatic site-specific cancer cell characteristics (e.g. cytokines), the organ-specific stroma, and by the origin of macrophages (e.g. Gr1^+^Ly6C^high^ inflammatory monocytes versus Ly6C^low^ resident monocytes [20]).

Importantly, our study provided evidence that MAM polarization is directly linked to the molecular characteristics that cancer cells acquire upon site-specific metastasis by (i) demonstrating an increased potential for M2 macrophage polarization by cancer cells that have metastasized to the brain parenchyma compared to the parental cancer cell line, and (ii) by demonstrating that the cancer cell-associated LTβ is functionally implicated in the polarization of parenchymal MAMs. The agonist-mediated activation of LTβR signaling in macrophages has been shown previously to inhibit their capability to upregulate NO and cytokine production upon re-stimulation with lipopolysaccharide *in vitro* [42]. This suggests that the downregulation of iNOS, TNFα and IFNγ in MAMs in our model may occur through a direct interaction between LTβ on cancer cells and the LTβR on MAMs. Alternatively, homotypic LTβ/LTβR interactions between cancer cells may alter their cytokine profiles, which may have an indirect effect on iNOS and inflammatory cytokines in MAMs. Since LTβ overexpression decreased the expression of only 3 out of 7 macrophage polarization markers, it is conceivable that MAM polarization is shaped by a combination of cytokines. Another cancer cell-derived factor that could be potentially involved in the polarization of MAMs in our model is CCL2 (~10-fold higher expression in parenchymal versus dural 4T1 cancer cell variants), which has been previously implicated in the M2 activation of human macrophages [54]. Moreover, a study in PyMT model implicated CCL2 in the attraction of inflammatory monocytes to the lungs were they differentiated into MAMs, while TAMs within the primary tumors were mainly derived from the resident monocytes [20]. Thus, due to the striking location-specific differences in CCL2 expression it is possible that dural and parenchymal MAMs in our model are derived from different monocyte populations, which may affect their polarization state. In conclusion, our data together with the current literature suggest that MAM phenotypes may be co-determined by the metastatic site-specific cancer cell features, the organ-specific stroma, as well as the origin of macrophages (Figure 6).

Based on the differences in molecular profiles of cancer cells and MAM phenotypes, metastases at the dura and within brain parenchyma are likely to respond differently to important emerging potential therapies. Major examples include immune checkpoint inhibitors (e.g. ipilimumab, nivolumab, pembrolizumab) [58], cytokine-targeting therapies (e.g. CCL2 inhibitors), or therapies targeting the two major transcription factors on which the cytokine-induced signaling pathways converge; NF-kB and Stat3 [28]. Moreover, macrophage repolarization strategies are being explored to add to the current cancer cell-targeting therapies [16, 33] and it would be important to determine the impact of metastatic location in this context. In conclusion, knowledge of distinct macrophage phenotypes in addition to cancer cell characteristics at individual metastatic sites, as exemplified for intracranial metastasis in our study, is expected to contribute to the development of improved therapies for patients with metastatic cancer.

## MATERIALS AND METHODS

### Ethics statement

Investigation has been conducted in accordance with the ethical standards and according to the Declaration of Helsinki and according to national and international guidelines and has been approved by the authors' institutional review board.

### Breast cancer cell lines

4T1 breast carcinoma was obtained from ATCC in January 2012. Human MDA-MB-231 breast cancer cells (originating from ATCC) were obtained from Dr. Felding-Habermann laboratory, The Scripps Research Institute, La Jolla, CA (TSRI) in January 2011 and validated by STR profiling in January 2016, confirming they are identical to the ATCC MDA-MB-231 line. PyMT 3503 cells [59, 60] were derived from spontaneous mammary fat pad tumors in PyMT mice and were kindly provided by Dr. Ruf from The Scripps Research Institute, La Jolla, CA in January 2011. All cell lines were confirmed to be Mycoplasma free in January 2016. All cell lines are also regularly inspected for their morphology by microscopy.

4T1 breast carcinoma and human MDA-MB-231 breast cancer cells were cultured as previously described [45]. PyMT cells were grown in L-15 medium w/o glucose supplemented with 10% FBS, glutamine, and 10 ug/mL insulin. For some experiments, cancer cells were stably transduced with Firefly luciferase- or GFP-expressing lentiviral vector [45, 61]. 4T1-Dura3 and 4T1-Par3 cell lines were obtained by pooling the biological triplicates of dura- and brain parenchyma-derived 4T1 cancer cell variants that underwent 3 rounds of *in vivo* selection. 4T1-Dura3-LTβ and 4T1-Dura3 control cell lines were generated by lentiviral transduction with pFUW-LTβ and empty pFUW vector, respectively.

### *In vivo* experiments

4T1 cells were grown in BALB/cAn mice, PyMT cells in C57Bl6/J mice and MDA-MB-231 cells in CB17/scid mice. All mice were 6–8 weeks old females and were purchased from Charles River Laboratories, UK or bred in house at St. James's Biological Services.

Cancer cells (1 × 10^5^ or 1 × 10^4^) were injected into the left external or internal carotid artery in a total volume of 50 μL, or implanted directly into the brain parenchyma as previously described [45, 61]. Non-invasive bioluminescence imaging was performed using IVIS Spectrum (PerkinElmer) [45, 61]. For site-specific selection of 4T1 cancer cell variants, 1 × 10^4^ parental 4T1 cells were injected into the internal carotid artery and two weeks later established dural and parenchymal metastases, respectively, were isolated. Following a short period of culturing and selection with 6-Thioguanine (4T1 cells are 6-Thioguanine resistant) the isolated cancer cells were subjected to a subsequent round of *in vivo* selection. Three different dural and parenchymal cell variants, respectively (biological triplicates) were established in parallel through 3 independent rounds of *in vivo* selection.

All procedures were approved by the University of Leeds Animal Welfare & Ethical Review Committee (AWERC), and performed under the approved UK Home Office project license in line with the Animal (Scientific Procedures) Act 1986 and in accordance with the UK National Cancer Research Institute Guidelines for the welfare of animals [62].

### Flow cytometry

Mice were perfused with saline. Dural brain metastases were micro-dissected from the skull and dissociated with trypsin, followed by collagenase/hyaluronidase treatment. For analysis of parenchymal metastases, the left half of the posterior 2/3 of the brain (containing cerebellum; the majority of bioluminescence signal localized to this part of the brain as seen in Figure 1A, Supplementary Figure S1B–S1C) was mechanically disrupted, followed by dissociation with collagenase/hyaluronidase. Myelin was removed using Myelin removal beads II (Miltenyi). Cells were blocked with 10% rat serum and subsequently stained for different hematopoietic markers prior to their analysis on BD LSRII Flow Cytometry Analyzer (Life Technologies).

Murine anti-Ly6G (1A8), anti-CD206 (C068C2), and anti-LTβR (5G11) were from Biolegend; anti-CD11b (M1/70) and anti-Ly6C (AL-21) were from BD Bioscience; anti-F4/80 (CI:A3-1) was from AbD Serotec; anti-CD3e (17A2), anti-Gr1 (RB6-8C5), anti-iNOS (CXNFT), anti-TNFα (MP6-XT22), and anti-IFNγ (XMG1.2) were from eBioscience; anti-CD45 (30F11), anti-MHCII (M5/114.15.2), and anti-CD11c (N418) were from Miltenyi Biotech; polyclonal anti-Arginase-1 antibody was from R&D Systems. The corresponding isotype control antibodies were from BioLegend, eBioscience, BD Bioscience or Miltenyi Biotech. Flow cytometry data were quantified using FACSDiva software.

### Immunofluorescence, H&E and verhoeff-van gieson staining

Mice were perfused with saline and 4% paraformaldehyde, followed by fixation in paraformaldehyde. The tissue was cut into free floating sections (brain parenchyma) or 10 μm frozen sections on slides (dural metastases). The same antibody clones were used as for flow cytometry. Images were acquired with AxioImager Z1 fluorescence microscope equipped with AxioCam MRc5 digital camera using AxioVision Rel. 4.7 software (Zeiss). Fixed whole heads were decalcified in Shandon TBD-2 decalcifier (Thermo Scientific) for 24 hours. Coronal sections of whole heads (10 um) were cut onto slides and stained with H&E or Verhoeff's Elastic Stain Kit (American MasterTech) according to the manufacturer's protocol.

### Gene expression and data analysis

Dura- and parenchyma-derived 4T1 cancer cell variants (biological triplicates obtained after 3 *in vivo* selection rounds) were harvested during exponential growth phase. RNA was isolated using RNAqueous Total RNA Isolation Kit (Ambion). The samples were processed using Amino Allyl MessageAmp II aRNA Amplification Kit (Ambion, AM1753). Gene expression analysis on Mouse Whole Genome OneArray MOA 2.1 and statistical data analysis was performed by Phalanx Biotech Group (Hsinchu, Taiwan). Briefly, the fluorescent signals on the arrays were scanned using Agilent Technology's DNA Microarray Scanner G2565B. The fluorescent intensities were analyzed with Rosetta Biosoftware and normalized using Rosetta Biosoftware's Rosetta Resolver System. Differential gene expression analysis between groups was performed using Rosetta's error model (http://bioinformatics.oxfordjournals.org/content/22/9/1111.full). A gene set enrichment analysis of pathways was performed using the differentially expressed gene lists as input into the DAVID website (http://david.abcc.ncifcrf.gov/). TFactS software was used to interrogate the regulation of transcription factors from the microarray data [34].

### qPCR

Taqman gene expression assays (mouse) were purchased from Life Technologies: *Ltβ* (Mm00434774_g1), *Ccl20* (Mm00444228_m1), *Ccl2* (Mm00441242_m1), *Cxcl1* (Mm04207460_m1), *Cxcl2* (Mm00436450_m1), *Csf2* (Mm01290062_m1), *Cxcr3* (Mm99999054_s1), *Gapdh* (Mm99999915_g1), *LtβR* (Mm00440235_m1),*Ltα* (Mm00440228_gH). Superscript III RT (Life Technologies) kit was used to synthesize cDNA from RNA that was isolated from pooled dura- and parenchyma-derived 4T1 cancer cell variants. The dCT values for individual probes were normalized to the GAPDH control.

### Western blot

Cancer cells were lysed in cell lysis buffer containing 1% Triton-X-100 and 0.1% SDS. Twenty μg of protein were loaded per lane. Detection was performed with rabbit polyclonal anti-LTβR antibody (Abcam). Anti-α-Tubulin (GeneTex) was used as loading control.

### LTβ lentiviral expression plasmid

The BamHI/XhoI fragment carrying murine LTβ ORF was excised from pCMV6-Entry plasmid (Origene) and sub-cloned into HpaI site of lentiviral vector pFUW [63] under Ubiquitin C promoter.

## SUPPLEMENTARY FIGURES AND TABLES


