# Supplementary material for: Metastatic site-specific polarization of macrophages in intracranial breast cancer metastases
Source: Oncotarget. 2016 May 18;7(27):41473–87. doi: 10.18632/oncotarget.9445 (PMC5173073; doi:10.18632/oncotarget.9445)
Supplement: Supplementary file 1 [file oncotarget-07-41473-s001.pdf]

Metastatic site-specific polarization of macrophages in intracranial breast cancer metastases

Supplementary Materials

Supplementary Table S1: Signaling pathways that were differentially regulated between the dura- and brain parenchyma-derived 4T1 cancer cell variants

Geneset name	# Genes in geneset (K)	# Genes in overlap (k)	k/K	*p* value
Cytokine-cytokine receptor interaction	244	24	0.09836	2.31E–07
Jak-STAT signaling pathway	152	12	0.07895	0.0034
p53 signaling pathway	69	7	0.10145	0.0124
Prostate cancer	90	8	0.08889	0.0128
Pathways in cancer	323	17	0.05263	0.0184
Focal adhesion	198	12	0.06061	0.0228
NOD-like receptor signaling pathway	62	6	0.09677	0.0293
Apoptosis	87	7	0.08046	0.0347

Supplementary Table S2: Differential expression of cytokine/cytokine receptors between the dura- and brain parenchyma-derived 4T1 cancer cell variants

Genes upregulated in parenchymal metastases			
Gene	Full name	Fold change	NF-kB1 target
Ltb	Lymphotoxin B	44.94	yes
Ccl20	Chemokine (C-C motif) ligand 20	26.91	yes
Ccl2	Chemokine (C-C motif) ligand 2	10.41	yes
Pdgfrb	Platelet derived growth factor receptor β polypeptide	9.78	
Cxcl1	Chemokine (C-X-C motif) ligand 1	8.40	yes
Csf2	Colony stimulating factor 2 (granulocyte-macrophage)	5.70	yes
Cxcl2	Chemokine (C-X-C motif) ligand 2	5.13	yes
Fas	Fas (TNF receptor superfamily member 6)	4.44	yes
Il3ra	Interleukin 3 receptor alpha chain	4.23	
Lif	Leukemia inhibitory factor	3.97	
Ccl7	Chemokine (C-C motif) ligand 7	3.76	
Cxcl5	Chemokine (C-X-C motif) ligand 5	3.41	yes
Inhba	Inhibin beta-A	3.10	yes
Il23a	Interleukin 23 alpha subunit p19	2.93	yes
Il1a	Interleukin 1 alpha	2.91	yes
Tnfsf15 (VEGI)	Tumor necrosis factor (ligand) superfamily member 15	2.89	yes
Il24	Interleukin 24	2.43	
Vegfa	Vascular endothelial growth factor A	2.31	

Pdgfa	Platelet derived growth factor alpha	2.06	
**Genes upregulated in dural metastases**			
**Gene**	**Full name**	**Fold change**	**NF-kB1 target**
Cxcl17	Chemokine (C-X-C motif) ligand 17	3.69	
Tnfrsf9	Tumor necrosis factor receptor superfamily member 9	2.00	yes
Flt1	FMS-like tyrosine kinase 1	2.00	
Il7	Interleukin 7	2.20	
Tnfsf10 (TRAIL)	Tumor necrosis factor (ligand) superfamily member 10	2.48	yes
Cxcr3	Chemokine (C-X-C motif) receptor 3	2.79	
Tnfsf8	Tumor necrosis factor (ligand) superfamily member 8	2.99	

Supplementary Table S3: Differential gene expression in parenchyma- versus dura-derived 4T1 cancer cells within the "NOD-like receptor signaling" pathway

Gene	Full name	Fold change
Map3k7	Mitogen-activated protein kinase kinase kinase 7	–2.07
Mefv	Mediterranean fever (Pyrin)	3.39
Ccl7	Chemokine (C-C motif) ligand 7	3.76
Cxcl2	Chemokine (C-X-C motif) ligand 2	5.13
Cxcl1	Chemokine (C-X-C motif) ligand 1	8.40
Ccl2	Chemokine (C-C motif) ligand 2	10.41

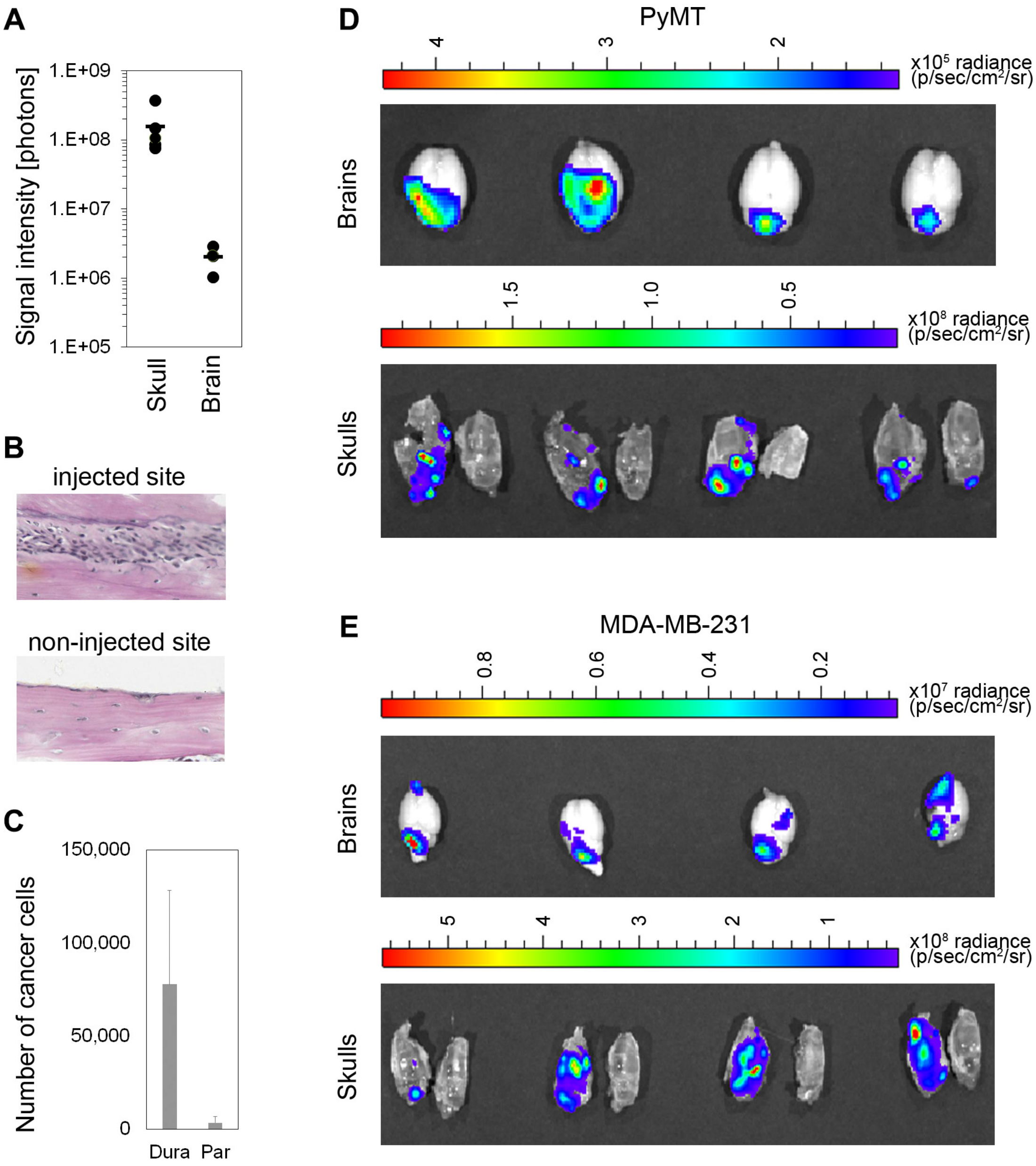

Supplementary Figure S1: Colonization of the skull/dura and brain parenchyma by breast cancer cells. (**A**) Distribution of cancer lesions between the skull/dura and the brain parenchyma was quantified upon the injection of 1 × 104 4T1 cancer cells into the internal carotid artery. (**B**) Cancer lesions within the left half of the skull (injected site) could be detected by H&E staining after administration of 4T1 cancer cells into the left internal carotid artery, while the contralateral half of the skull was devoid of cancer lesions. (**C**) Quantification of GFP-tagged cancer cells within brain parenchyma and the dura upon their administration into the internal carotid artery by flow cytometry. Total numbers of cancer cells per sample are shown. Significant differences were determined using two-tailed *T*-test with unequal variance ($p = 0.04$); $n = 4$; error bars represent SD. (**D** and **E**) Distribution of cancer lesions between the skull/dura and the brain parenchyma was analyzed by *ex vivo* bioluminescence imaging at 16 and 45 days post-cancer cell injection into the internal carotid artery using PyMT (D) and MDA-MB-231 cancer cell lines (E), respectively.

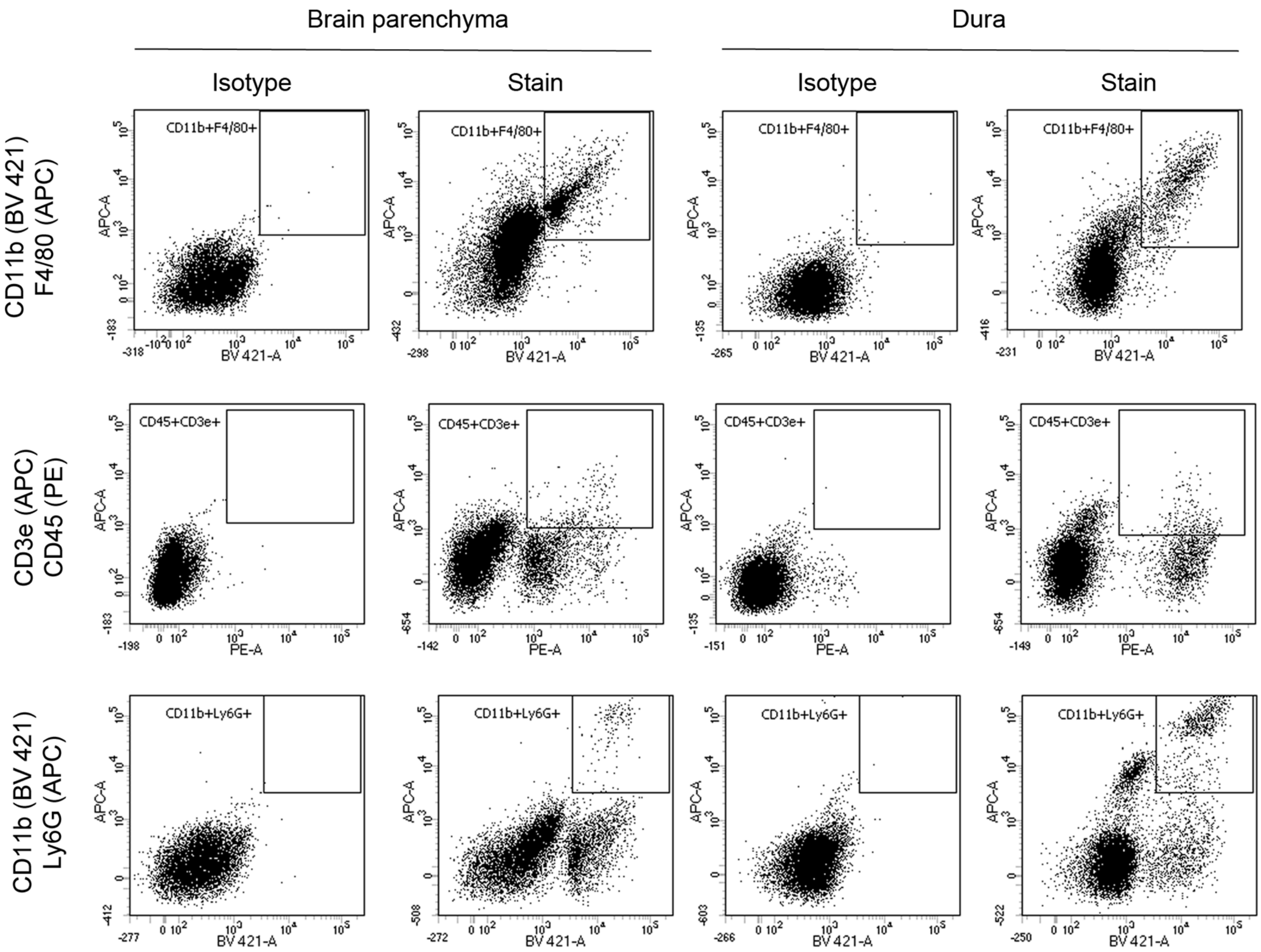

Supplementary Figure S2: Analysis of immune cells infiltrating intracranial 4T1 metastases by flow cytometry. Examples of dot plots of flow cytometry analysis shown in Figure 2A, including the isotype control staining.

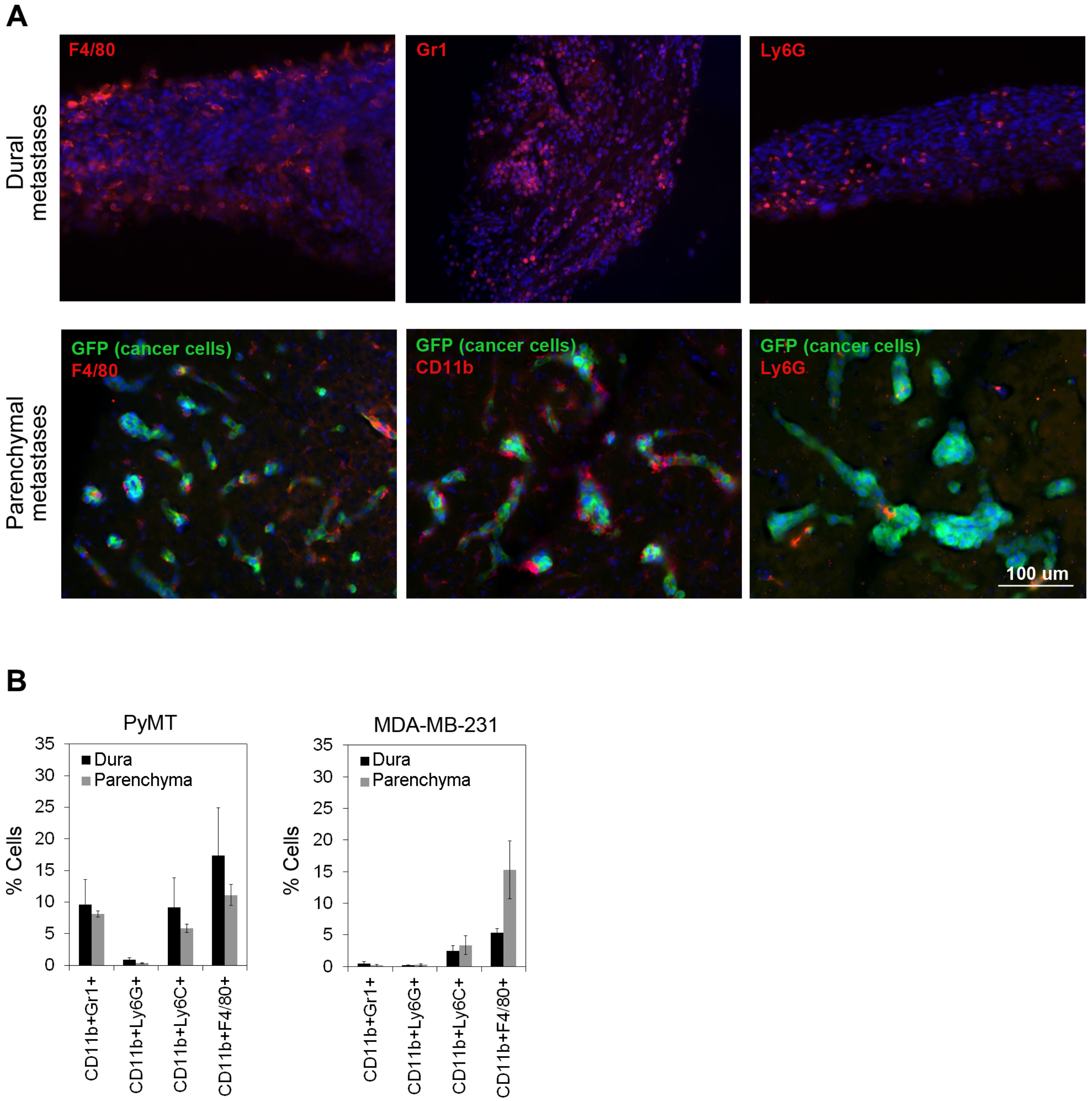

Supplementary Figure S3: Infiltrating immune cells in dural and parenchymal metastases. (**A**) Immunofluorescence staining for macrophages (F4/80), myeloid derived suppressor cells (Gr1), neutrophils (Ly-6G) and/or myeloid cells (CD11b) in dural (top) and parenchymal (bottom) 4T1 metastases. (**B**) The infiltration of immune cells into dural (dura) and parenchymal metastases (parenchyma) in PyMT and MDA-MB-231 breast cancer models was quantified by flow cytometry.

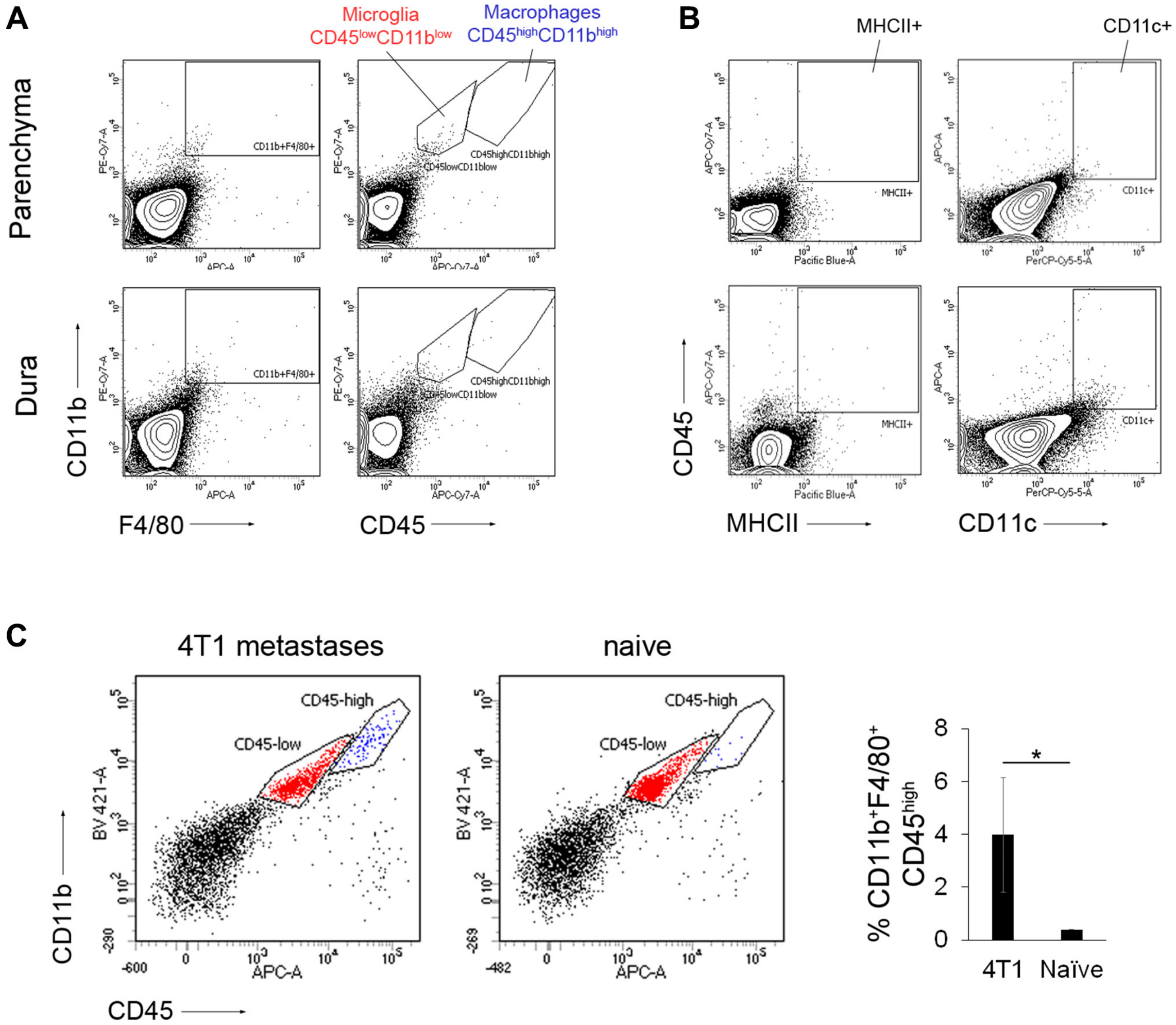

Supplementary Figure S4: Infiltration of microglia and macrophages into intracranial 4T1 metastases and into naïve brain. (**A**) Isotype control staining for flow cytometry analysis shown in Figure 2B. (**B**) Isotype control staining for flow cytometry analysis shown in Figure 2D. (**C**) Infiltration of macrophages (CD11b^{+}F4/80^{+}CD45^{high}) into the brain parenchyma of naïve BALB/c mice as compared to the 4T1 metastases-bearing brain. Quantification is shown on the right. Statistical significance was determined using two-tailed Student's *T* test.

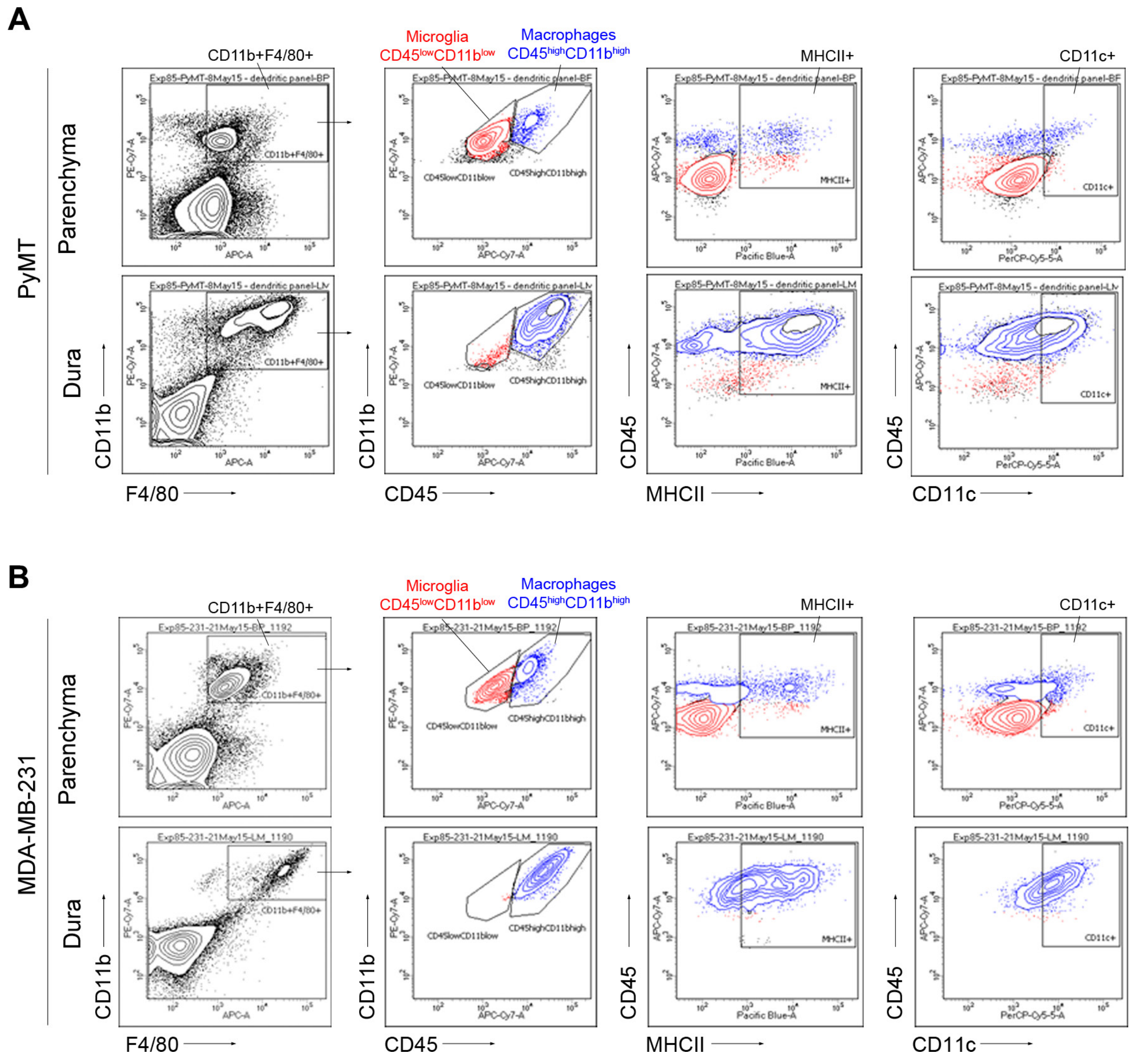

Supplementary Figure S5: Expression of antigen-presenting cell markers in dural and parenchymal MAMs isolated from PyMT (A) and MDA-MB-231 models (B). Representative flow cytometry analysis of microglia ($CD45^{low}CD11^{blow}$ cells within the $CD11b^{+}F4/80^{+}$ gate; red) and macrophages ($CD45^{high}CD11b^{high}$ cells within the $CD11b^{+}F4/80^{+}$ gate; blue) within parenchymal (top) and dural lesions (bottom) is shown in the left two panels. Representative flow cytometry analysis of MHCII and CD11c expression within microglia and macrophages (gated on $CD11b^{+}F4/80^{+}$ cells) is shown in the right two panels.

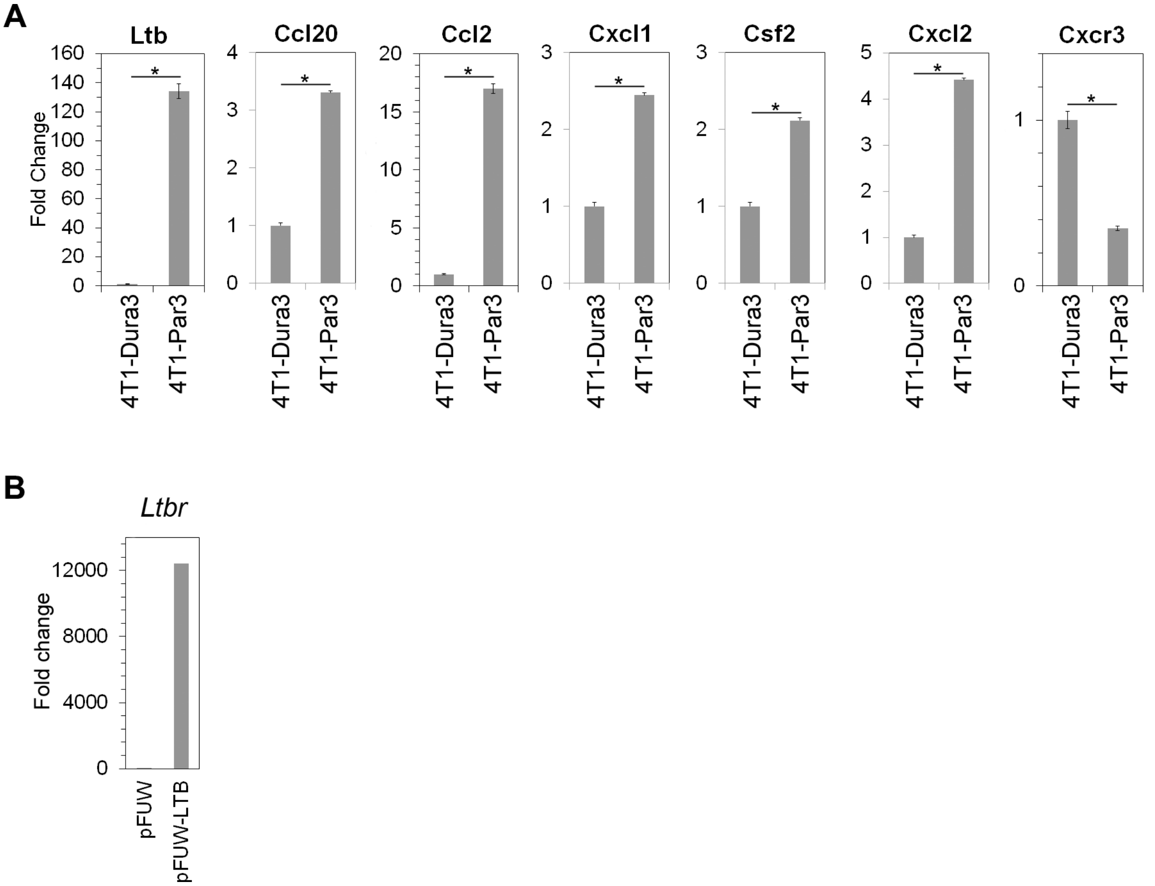

Supplementary Figure S6: Gene expression as detected by qRT-PCR. (**A**) The expression of cytokines whose expression was most strongly altered between the 4T1-Par3 and 4T1-Dura3 cancer cell lines was analyzed by qRT-PCR. Significant differences were determined using two-tailed *T*-test with unequal variance ($p \leq 0.05$); $n = 3$; error bars represent SD. (**B**) Overexpression of LTβ in the 4T1-Dura-3 cell line (pFUW-LTB). *Ltβ* mRNA levels were quantified in comparison to the cells transduced with empty pFUW vector.

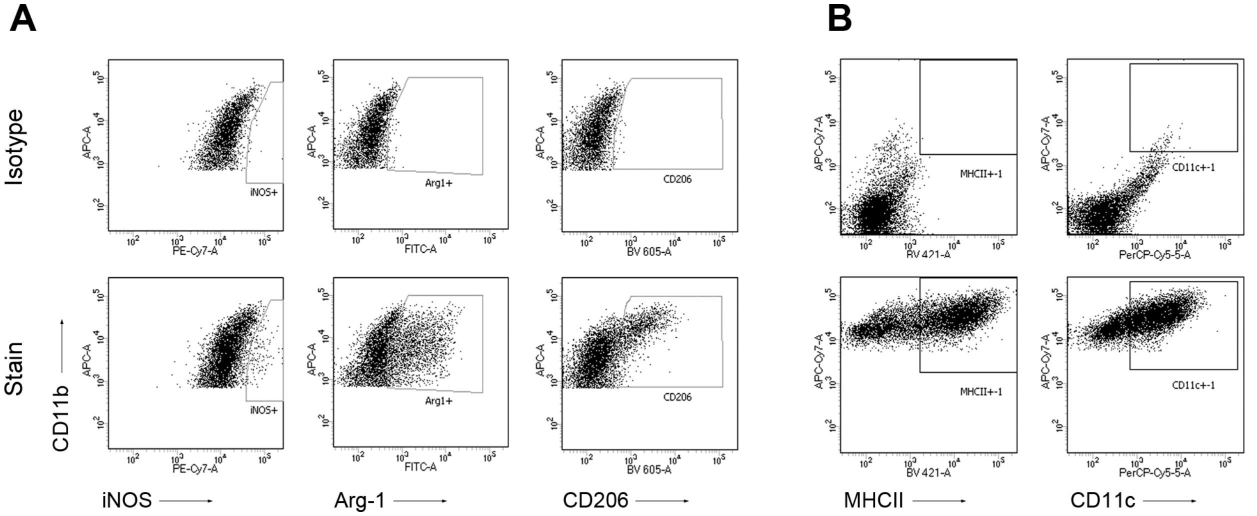

Supplementary Figure S7: Analysis of macrophage polarization by flow cytometry. Examples of dot plots showing CD11b^{+}F4/80^{+}Gr1-CD45^{high} cell population within dural metastases (corresponding to the data shown in Figure 4C, D). (**A**) Isotype (top row): stained for anti-CD11b, anti-F4/80, anti-Gr1, anti-CD45 plus the isotype control corresponding to one of the 3 markers (iNOS, Arg-1, CD206). Stain (bottom row): stained for anti-CD11b, anti-F4/80, anti-Gr1, anti-CD45 plus one of the 3 markers (iNOS, Arg-1, CD206). (**B**) Isotype (top row): stained with isotype control antibodies only. Stain (bottom row): stained for anti-CD11b, anti-F4/80, anti-Gr1, anti-CD45 plus MHCII or CD11c.
